# Impact of imidacloprid on new queens of imported fire ants, *Solenopsis invicta* (Hymenoptera: Formicidae)

**DOI:** 10.1038/srep17938

**Published:** 2015-12-08

**Authors:** Lei Wang, Ling Zeng, Jian Chen

**Affiliations:** 1 College of Agriculture, South China Agricultural University, 483 Wushan Road, Guangzhou, Guangdong, 510642, P. R. China; 2 National Biological Control Laboratory, Southeast Area, Agriculture Research Service, United States Department of Agriculture, 59 Lee Road, Stoneville, MS 38776

## Abstract

Neonicotinoid insecticides are commonly used in managing pest insects, including the imported fire ant, *Solenopsis invicta* Buren. There is increasing evidence that neonicotinoid insecticides at sublethal concentrations have profound effects on social insects. However, the sublethal effect of neonicotinoids on *S. invicta* has never been investigated. In this study, the newly mated queens were fed with water containing 0.01 or 0.25 μg/ml imidacloprid. Imidacloprid at both concentrations did not cause any increase in queen mortality during the founding stage; however, it significantly reduced queens’ brood tending ability. In the 0.25 μg/ml imidacloprid treatment, the time to larval emergence was significantly delayed and no pupae or adult workers were produced. This study provides clear evidence that imidacloprid at sublethal concentrations has a significant detrimental impact on *S. invicta* queens and the development of incipient colonies.

Neonicotinoid insecticides are used worldwide for controlling insect pests^1^,^2^,^3^. The effect of neonicotinoid insecticides on non-target organisms has been well documented^4^,^5^,^6^,^7^,^8^. Neonicotinoid residues have been found in soils, water, crops^9^ and even in bee pollen^8^. There is increasing evidence that neonicotinoid insecticides at sublethal concentrations have profound effects on insects. They affect learning, memory and orientation in honeybees and brood production, larval eclosion, colony growth rate and the number of queens reared in bumblebees^10^. Neonicotinoid insecticides at low doses also affected the behavioral responses of the black cutworm, *Agrotis ipsilon* (Hufnagel), to sex pheromone^11^. Neonicotinoid insecticides at sublethal levels also have complicated effect on ants. For example, imidacloprid reduced the grooming behavior of *Acromyrmex subterraneus subterraneus* (Forel)^12^. Exposure to sublethal neonicotinoids increased the aggressiveness of the Argentine ant, *Linepithema humile* (Mayr), which increased their survival in the confrontation with native Southern ant, *Monomorium antarcticum* (Smith)^13^, which led to an interesting hypothesis that neonicotinoids may facilitate the spread of invasive species.

Neonicotinoids at sublethal concentrations also have species-specific effects on the fecundity of arthropods. The fecundity of two-spotted spider mites and *Amblyseius victoriensis* (Womersley) was increased after being treated with imidacloprid^14^,^15^. Exposure to sublethal concentrations of imidacloprid and azadirachtin stimulated the reproduction of green peach aphid, *Myzus persicae* (Sulzer)^16^. In contrast, imidacloprid at sublethal concentrations reduced the fecundity of brown planthopper, *Nilaparvata lugens* (Stål)^17^. Neonicotinoids have been shown to have a negative effect on the fecundity of social insects. Chronic dietary neonicotinoid exposure has severe detrimental effects on solitary bee offspring production and causes a male-biased offspring sex ratio^18^. Imidacloprid significantly reduced bumble bee brood production, colony growth, and queen production^19^,^20^,^21^. However, a recovery of brood production in bumble bee colonies during an ‘off-imidacloprid’ period was observed^22^.

Ants are important species in the terrestrial environment^23^. They are crucial for the maintenance and functioning of many ecosystems by providing a variety of ecosystem services^24^. Disrupting those services will inevitably affect the health of various ecosystems. Because of their wide distribution in nature, ants may have multiple routes to contact neonicotinoids insecticides. However, few studies have been conducted to assess the sublethal effects of neonicotinoids on ant behavior and colony fitness^12^,^13^. It was found that imidacloprid at sublethal concentrations have species-specific effects on ant reproduction. Brood production of Southern ant, *M. antarcticum*, was not affected after exposure to 1.0 μg/ml imidacloprid, but brood production of Argentine ant *L. humile* was significantly reduced^13^.

The red imported fire ant*, Solenopsis invicta* Buren is a globally distributed invasive ant^25^ and one of the most well-studied ant species^26^. Neonicotinoid insecticides are used in managing pest ants, including *S. invicta*^27^. However, the sublethal effect of neonicotinoids on *S. invicta* has never been investigated. In this study, we examined the effect of the imidacloprid at sublethal concentrations on fire ant queens and colony development. Colonies at founding stage provide a good opportunity to conduct such research, because of the ease of colony maintenance and observation. Newly mated queens can be easily collected from the field and a colony can be started using an artificial queen chamber without any food. Before the first batch of workers emerges, queens have to perform all the tasks needed for the development of the colony, which provides a window to observe the effect of imidacloprid on queen behaviors. Individual queens were provided with distilled water containing imidacloprid in a transparent queen chamber and the effect of the imidacloprid on queen mortality, response to light, egg and brood tending behavior, and development of the incipient colonies was assessed.

## Results

### Effect of imidacloprid on queen reproduction and development of incipient colonies

**Figure 1:**
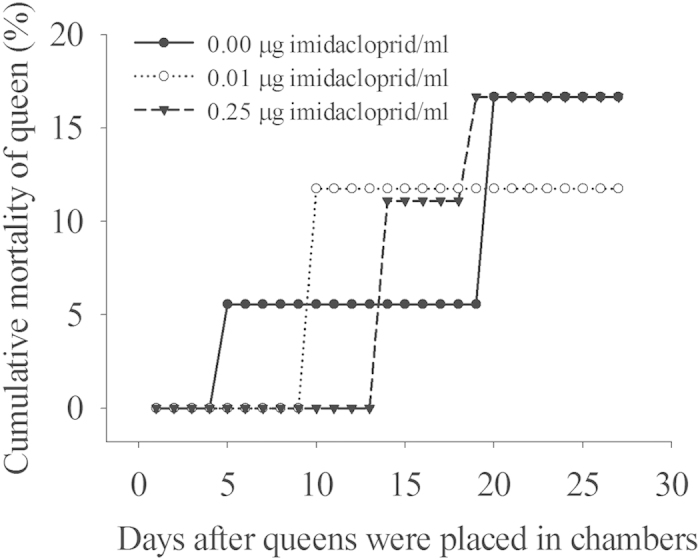
Cumulative mortalities 27 days after queens were placed in the artificial queen chambers that were supplied with water containing different concentrations of imidacloprid (the cumulative queen mortality after 27days, Pearson chi-squre test, *X*^2^ = 0.22, *df* = 2, *P* = 0.90).

Twenty-seven days after collection, queen mortality was 16.67%, 11.76%, and 16.67%, for the control, 0.01 μg/ml, and 0.25 μg/ml imidacloprid treatments, respectively (Fig. 1). Imidacloprid at both 0.01 μg/ml and 0.25 μg/ml did not cause an increase in queen mortality (Pearson chi-squre test, *X*^2^ = 0.22, *df* = 2, *P* = 0.90). All queens in each treatment produced eggs (Table 1). In the control, 17 of 18 queens produced eggs. In the control, 77.78% of queens produced pupae. In the 0.01 μg/ml imidacloprid treatment, only 64.71% of queens produced pupae. In 0.25 μg/ml imidacloprid treatment, no queens were able to produce pupae (Table 1). It was observed that moldy eggs appeared in 10 of 18 replicates in the 0.25 μg/ml imidacloprid treatment 8 days after the experiment started. Afterward, the number of replicates with moldy eggs kept increasing. In the 0.01 μg/ml imidacloprid treatment, only 2 of 17 replicates had moldy eggs at the 8^th^ day and they disappeared in two or three days later. In contrast, no moldy eggs were observed in the control. In the control, 14 of 18 queens produce workers and after 6 weeks, 11 of queens survived. In the 0.01 μg/ml imidacloprid treatment, 11 of 17 queens produced workers, and 8 of queens survived after 6 weeks. However, no queens produced pupae or workers in the 0.25 μg/ml imidacloprid treatment (Table 1). There was no significant difference in preoviposition duration among the three treatments (Table 2, LSD test, *F*_2,48_ = 0.67, *P* = 0.52). There was no significant difference in egg duration between the control and the 0.01 μg/ml imidacloprid treatment (Mann-Whitney test, U = 64, *P* = 0.075); however, egg duration in the 0.25 μg/ml treatment was significantly longer than that in both the control (Mann-Whitney test, U = 38, *P* = 0.001) and 0.01 μg/ml imidacloprid treatments (Mann-Whitney test, U = 45.5, *P* = 0.019). There was no significant difference in larval, pupal, and egg-to-adult duration between the control and the 0.01 μg/ml imidacloprid treatment (independent t test, larvae duration: *t* = 0.049, *df* = 24, *P* = 0.962; pupae duration: *t* = 0.791, *df* = 23, *P* = 0.437; eggs-to-adult duration: *t* = −1.576, *df* = 23, *P* = 0.129).

**Figure 2:**
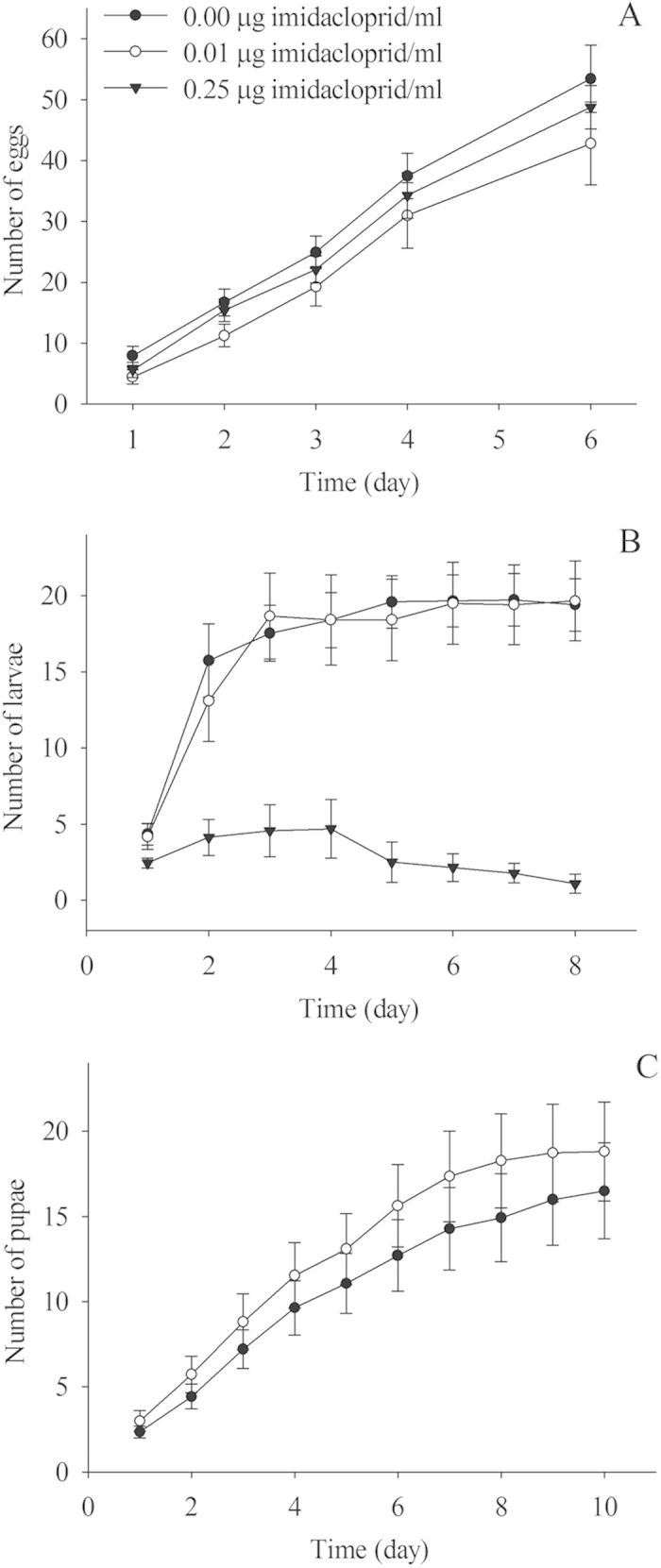
Effect of imidacloprid on the production of eggs, larvae and pupae. No pupae were produced in the 0.25 μg/ml imidacloprid treatment. Day 1 was defined as the day when the first ant of the corresponding stage emerged.

There was a significant difference in the number of eggs between the control and imidacloprid treatments (Fig. 2A, *F*_2,4_ = 4.24, *P* = 0.016). There were significantly fewer eggs in the 0.01 μg/ml imidacloprid treatment than in the control; however, the number of eggs in the 0.25 μg/ml imidacloprid treatment was not significantly different from both the control and the 0.01 μg/ml imidacloprid treatments. There was a significant difference in the number of larvae between the control and imidacloprid treatments before pupae emergence (Fig. 2B, *F*_2,7_ = 114.68, *P* < 0.0001). There was no significant difference in the number of larvae between the control and the 0.01 μg/ml imidacloprid treatments, but significantly fewer larvae were recorded in the 0.25 μg/ml imidacloprid treatment than in the control and the 0.01 μg/ml imidacloprid treatments. Just prior to adult emergence, significantly more pupae were recorded in 0.01 μg/ml imidacloprid treatment than in the controls (Fig. 2C, *F*_1,9_ = 5.53, *P* = 0.020).

Water consumption and imidacloprid intake are summarized in Supplementary Figure 1. Total water consumption 27 days after queens were placed in queen chambers was not significantly different between treatments and the control (LSD test, *F*_2,42_ = 2.68, *P* = 0.080). And the average imidacloprid intake in the 0.01 and 0.25 μg/ml treatments was 9.94 and 233.37 ng/queen, respectively (Kruskal test, *H* = 40.69, *df* = 2, *P* < 0.0001). The number of brood (larvae and pupae) and workers in the incipient colonies was counted when the first group of workers emerged. There was no significant difference in the number of brood and workers and total population sizes between the control and the 0.01 μg/ml imidacloprid treatment (Table 3; workers, *t* = 1.278, *df* = 23, *P* = 0.214; brood, *t* = −0.220, *df* = 23, *P* = 0.828; total population, *t* = −0.174, *df* = 23, *P* = 0.863). After 6 weeks, the number of brood and workers in each colony that had a live queen was recorded. The number of brood, workers, and the total population were also not significantly different between the control and the 0.01 μg/ml imidacloprid treatment (Table 3; workers, *t* = 0.154, *df* = 16.36, *P* = 0.879; brood, *t* = 0.897, *df* = 15.59, *P* = 0.383; total population, *t* = 0.774, *df* = 15.60, *P* = 0.450).

The consumption of water and sugar water, and imidacloprid intake during the 6 weeks following worker emergence are summarized in Supplementary Figure 2. Water consumption was not significantly different between the control and the 0.01 μg/ml imidacloprid treatment (independent *t* test, *t* = −0.572, *df* = 17, *P* = 0.575). However, colonies in the 0.01 μg/ml imidacloprid treatment consumed more sugar water than in the control (independent *t* test, *t* = −4.39, *df* = 16.70, *P* < 0.0001). Average imidaclorpid intake in the 0.01 μg/ml imidacloprid treatment was 47.09 ng/colony.

### Effect of imidacloprid on queen brood tending behavior

**Figure 3:**
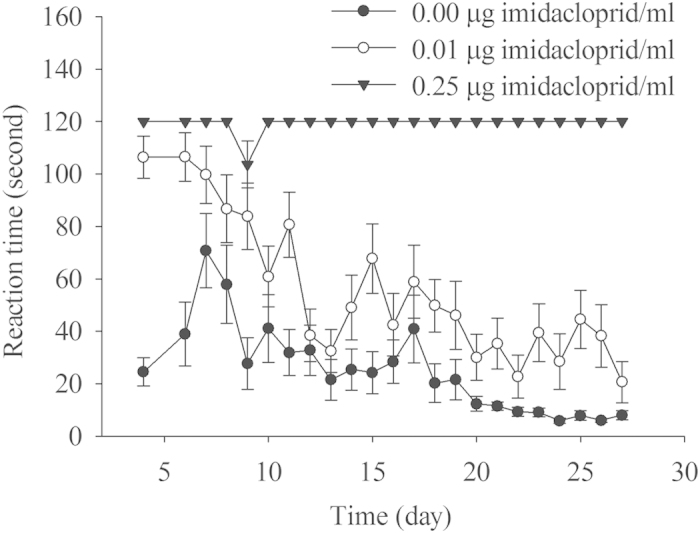
The effect of imidacloprid on the queen reaction time to light with 15,140 lux intensity. The reaction was defined as the time period from when the queen was exposed to the light to when the queen lifted the first brood clump. If a queen did not move the brood within 2 min, the time was recoded as 120 sec.

**Figure 4:**
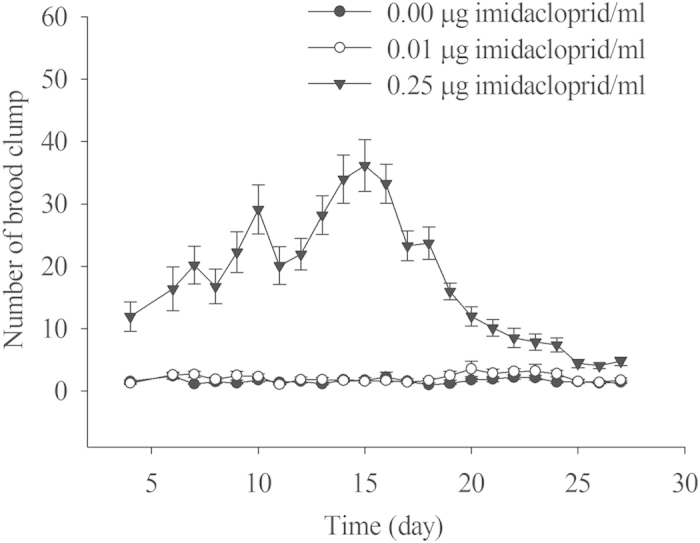
The effect of imidacloprid on the number of egg and brood clumps.

The reaction times of fire ant queens to light with 15,140 lux intensity (from when the queen was exposed to light to when the queen lifted the brood clump) are summarized in Fig. 3. A dose-dependent increase in reaction time was found. The reaction time in the control was generally below 40 seconds, which was significantly shorter than that for the imidacloprid treatments (*F*_2,21_ = 767.84, *P* < 0.0001). Data on brood clumps are summarized in Fig. 4. The number of brood clumps in the 0.25 μg/ml imidacloprid treatment was significantly greater than those in both the control and 0.01 μg/ml imidacloprid treatment (*F*_2,21_ = 490.24, *P* < 0.0001). There was no significant difference in number of brood clumps between the control and the 0.01 μg/ml imidacloprid treatment.

## Discussion

The effect of neonicotinoid pesticides on honeybee behaviors has been well documented. They affect the olfactory learning and memory^28^,^29^,^30^, visual learning^31^, gustatory sensitivity to sucrose, ability to perform the waggle dance^32^, and motor function^33^. Due to the complexity of a honeybee society, subtle influence on behaviors is believed to affect normal function of a honeybee colony in the field^33^. A recent study demonstrated that exposure to field-realistic concentrations of neonicotinoid pesticides severely affected the queen development of western honey bees, *Apis mellifera*^34^.

Imidacloprid is a widely used insecticide; its residue was detected at various levels in soils, waterways, and plants^9^. For example, 0.12 to 0.22 mg/kg imidacloprid was detected in soils in a vineyard after harvest at recommended and double recommended dosages of insecticide applications^35^. In a study on the residue of imidacloprid in vegetables, fruits, and water samples, it was found that the highest and lowest imidacloprid concentrations were found in eggplant (0.46 mg/kg) and green beans (0.08 mg/kg), respectively^36^. Neonicotinoids are commonly used in seed coatings to control insect pests in a variety of crops^37^. Red imported fire ants are known to feed upon planted field crop seeds^38^. As a soil dwelling ant species and crop seed pest, *S. invicta* may have various routes of exposure to imidacloprid. This study provides the first evidence that imidacloprid at sublethal concentrations has a significant impact on red imported fire ant queens. Although imidacloprid at two tested concentrations didn’t cause any mortality to the queens during the founding stage, it significantly changed their sensitivity to light and their brood tending behavior. These changes may be detrimental to the development of the colonies, which is evidenced by the complete failure of brood production in the 0.25 μg/ml imidacloprid treatment.

Immediately after the nuptial flight, queens shed their wings and 1–5 queens construct a small nest^39^. Within 24 hours the queen(s) closes the entrance and raises the first brood in this small nest. Minims, the first batch of workers with much smaller body sizes, reopen the nest and begin to forage for the colony. Before the emergence of the minims, queens have to perform brood tending tasks by themselves, so any negative effect on queen brood tending behavior may have a great impact on colony development. In a healthy fire ant colony, eggs and larvae cling to each other and are stored and transported in clumps, which is achieved by either coating materials on eggs and 1^st^ and 2nd instar larvae or by hairs on 3nd and 4^th^ instar larvae^40^. Before workers appear, queens constantly groom the eggs and young larvae. The adhesive coating is probably either applied or at least kept moist by the adults^40^. Imidacloprid might have reduced the queen’s ability to produce and/or secrete the coating material or the ability to keep those materials moist, which resulted in the increased number of brood clumps in the colony. In such a situation, it became a much harder task for the queen to tend egg and brood. Eggs in the imidacloprid treatments might receive significantly less tending from the queen than those in the controls. Untended eggs always become moldy^41^. This may be the reason why moldy eggs were only found in imidacloprid treatments. Suppression of social behaviors by nicotinoid insecticides was also found in termites, the compromised social behaviors synergized the impact of entomopathogens on the termites^42^.

Queens in the 0.25 μg/ml imidacloprid treatments produced more eggs than those in the 0.01 μg/ml imidacloprid treatment. Since the differences between the control and imidacloprid treatments were not statistically significant, no conclusion could be made about the effect of imidacloprid on egg production. In the 0.25 μg/ml imidacloprid treatment, the time to larval emergence was significantly delayed compared to both the control and the 0.01 μg/ml imidacloprid treatment. Some eggs failed to hatch and became moldy in the 0.25 μg/ml imidacloprid treatment, which might cause the delay of larval emergence. Number of larvae per colony before pupal emergence was significantly lower in the 0.25 μg/ml imidacloprid treatment than in both the control and the 0.01 μg/ml imidacloprid treatment. Newly mated queens of *S. invicta* lay embryonated eggs in first six days after the nuptial flight, but afterwards they lay only trophic eggs as a food source for new hatched larvae until the first batch of workers emerges^43^. In addition to the higher mortality of embryonated eggs, moldy eggs might also indicate that less food was available to the larvae. This may explain why colonies in the 0.25 μg/ml imidacloprid treatment failed to produce pupae and adult workers.

Queens in the imidacloprid treatments responded to light significantly more slowly than those in the control. An earlier experiment showed that the walking speed of workers was also significantly decreased in the 0.01 μg/ml imidacloprid treatment (data are not reported here). Fire ant incipient colonies face fierce competition from conspecifics and other ant species. In the field, only 25% of founding colonies survived in the early incipient stage because of brood raiding^44^, attacks from other mature colonies^45^ and other ant species^46^. Imidacloprid at low doses may reduce ant competitive ability by slowing their responsiveness.

In haplometrotic fire ant colonies (newly-mated queens start new colonies individually), 3.4% early queen mortality was reported before worker eclosion (8 out of 252 queens died)^47^. In this study, 3 out 18 queens died in the control (16.67%). This discrepancy in queen mortality might be just due to the huge difference in sample sizes between these two studies.

Water consumption of newly founded colonies was not significantly different between the control and treatments before the first group of workers emerged. However, after the first group of workers emerged, the incipient colonies in the 0.01 μg/ml imidacloprid treatment consumed significantly more sugar water than those in the control, but water consumption was not significantly different. This indicates that imidacloprid may have different impacts on the feeding behavior of different castes. Imidacloprid intake at this level may stimulate sugar consumption by workers. Whether imidacloprid intake increased the total food consumption could not be determined because data on the cricket (food) consumption were not recorded. Interestingly, it was recently reported that honeybee, *Apis mellifera* Linnaeus, and the buff-tailed bumblebee, *Bombus terrestris* Linnaeus, do not avoid nectar-relevant concentrations of imidacloprid in food^48^. Moreover, bees of both species prefer to eat more of sucrose solutions laced with imidacloprid than sucrose alone. It will be interesting to see whether newly mated *S. invicta* queens show preference to imidacloprid-containing soil while they are constructing their queen chambers.

The results from this study may help us develop new approaches for more effective utilization of neonicotinoid pesticides in fire ant management. On the other hand, the results can also serve as an alert for the potential detrimental impact of neonicotinoid insecticide residuals on beneficial ants.

## Materials and Methods

### Imidacloprid

Imidacloprid (>99.9% purity, Sigma-Aldrich, St. Louis, Missouri, USA) was directly dissolved in sterile distilled water. Based on our previous studies on the sublethal effect of imidacloprid on *S. invicta* behavior (Wang, unpublished), two concentrations of imidacloprid, 0.25 and 0.01 μg/ml, were selected for this study. Solutions were freshly prepared before each experiment.

### Queen collection and the artificial queen chamber

Newly mated fire ant queens were collected on the grounds of a plaza in Greenville, Mississippi, USA on September, 19, 2014. Individual queens were placed in a queen chamber, a compartmentalized glass tube (10 cm length × 1.2 cm diameter) (Supplementary Figure 3). Two compartments were separated by a sterile cotton ball. The upper compartment housed the queen. The bottom compartment was filled with either an imidacloprid water solution or distilled water. The tube was plugged by another cotton ball to prevent the queen from escaping. In order to calculate the water consumption, the weight of each chamber with a queen was then recorded. All queens were maintained under dark condition in a controlled rearing broom at 28.0 °C and 60.0% RH.

### Effects of imidacloprid on queen reproduction and development of incipient colonies

Seventeen queens were used in the treatment with 0.01 μg/ml imidacloprid and 18 queens were used in the treatment with 0.25 μg/ml imidacloprid and in the control. Queen status was checked every day. The number of eggs, pupae, larvae and workers was recorded daily for each queen. Preoviposition duration was defined here as the time period from when the queen was collected to when the queen laid her first egg. Egg duration was the time period from when the first egg was produced to when the first larva emerged. The larval duration was the time period from when the first larva emerged to when the first pupa appeared. The pupal duration was the time period from when the first pupa emerged to when the first worker emerged. After the first group of workers emerged, the chamber was weighed again and then placed in a small plastic box (15 cm length × 9 cm width × 7 cm height). The inner walls of the box were coated with Fluon. The queen chamber was opened by removing the cotton ball on the top. Sugar water (5% w/w sugar) and frozen adult house crickets, *Acheta domesticus (Linnaeus)* (Armstrong’s Cricket Farm, Glennville, GA), were supplied as food sources. Water containing imidacloprid at the same concentration as that in the queen chamber was also provided in a test tube. The queen status and the number of eggs, larvae, pupae, and workers were recorded for each colony. The experiment ended 6 weeks after the queen chamber was opened. The consumption of water, sugar water and imidacloprid was calculated for each surviving queen. The daily consumption was adjusted for the natural water evaporation, which was estimated by using the same setup without ants. The verification of ant species for each colony was confirmed using the cuticular hydrocarbon profile of the queen.

### Effects of imidacloprid on queen brood tending behavior

Queens were observed under a Nikon SMZ1500 zoom stereomicroscope. In a preliminary test, we observed that brood of *S. invicta* was always in clumps and the queen moved brood clumps around in the chamber in response to the light from a high intensity illuminator (Nikon Instruments Inc. Lewisville, TX). The number of brood clumps was recorded daily for each queen. Reaction time to light was recorded for each queen. The reaction time was defined as the time period from when the chamber was put under the microscope to when queen start moving brood. If a queen did not move brood within 2 min, she was labeled as “no reaction”. Light intensity was 15,140 lux, which was measured using EasyView^TM^ 30 light meter (Extech Instruments, Nashua, NH). Daily data collection on number of brood clumps and queen reaction times began on the 4^th^ day after newly mated queens were collected, because that was the time for most of queens to lay eggs. Data collection ended 27^th^ days after the queens were collected, when adult workers began emerging.

### Statistical analysis

All data were tested for normal distribution by the Shapiro-Wilk test and for homogeneity of variances by Levene’s test. Pearson chi-square test was used to compare the queen mortalities in the control and two imidacloprid treatments. Egg numbers were compared using the LSD test. Egg duration was compared between treatments with the Kruskall-Wallis ANOVA and Mann-Whitney U test. Independent *t* tests were performed to compare larvae duration, pupae duration, and duration from eggs to adult workers between the control and the 0.01 μg/ml imidacloprid treatment. Reaction time to light, number of brood clumps, and the numbers of eggs, larvae and pupae were compared using generalized linear model (GLM). All statistical analyses were performed in SPSS version 18.0 (SPSS inc., Chicago, IL, USA).

## Additional Information

**How to cite this article**: Wang, L. *et al.* Impact of imidacloprid on new queens of imported fire ants, *Solenopsis invicta* (Hymenoptera: Formicidae). *Sci. Rep.* **5**, 17938; doi: 10.1038/srep17938 (2015).

## Supplementary Material

Supplementary Information

## Figures and Tables

**Table 1:** The number of queens that were capable of producing offspring to different stages and survival of the incipient colonies.

Imidacloprid (μg/ml)	Initial number of queens	Queens producing offspring				Colony survival rate (%)*
		Eggs	Larvae	Pupae	Workers	
0.00	18	17	16	14	14	61.11
0.01	17	17	12	11	11	47.06
0.25	18	18	16	0	0	0.00

^*^Survival rate 6 weeks after the queen chamber was opened.

**Table 2:** Effect of imidacloprid on the duration of each developmental stage.

Imidacloprid (μg/ml)	Duration (day, mean ± SE)				Egg-to-adult
	Preoviposition	Egg	Larval	Pupal	
Control	1.94 ± 0.46	6.65 ± 0.23	9.93 ± 0.33	10.50 ± 0.14	26.78 ± 0.38
0.01	1.88 ± 0.34	7.33 ± 0.28	9.91 ± 0.37	10.73 ± 0.27	27.73 ± 0.47
0.25	1.44 ± 0.12	9.27 ± 0.59	–	–	–

**Table 3:** Effect of imidacloprid on the sizes of incipient ant colonies.

Imidacloprid (μg/ml)	When workers first emerged			Six weeks after the first emergence of workers		
	Brood	Workers	Total	Brood	Workers	Total
0.00	54.07 ± 9.71	2.14 ± 0.33	56.21 ± 9.94	64.00 ± 17.43	286.64 ± 78.46	350.64 ± 94.45
0.01	56.73 ± 5.65	1.64 ± 0.15	58.36 ± 5.72	60.75 ± 11.80	204.62 ± 46.94	265.38 ± 56.66

There was no significant difference between control and 0.01 μg/ml imidacloprid treatment for each category (*t*-test, *P* > 0.05).
