# Supplementary material for: Impact of imidacloprid on new queens of imported fire ants, Solenopsis invicta (Hymenoptera: Formicidae)
Source: Sci Rep. 2015 Dec 8;5:17938. doi: 10.1038/srep17938 (PMC4672302; doi:10.1038/srep17938)
Supplement: Supplementary Information [file srep17938-s1.docx]

**Impact of imidacloprid on new queens of imported fire ants, *Solenopsis invicta*** **(Hymenoptera: Formicidae)**

Lei Wang, Ling Zeng and Jian Chen

**Supplementary information**

**
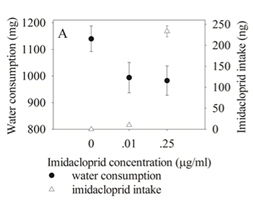
**

**Supplementary Figure 1.** Water consumption and imidacloprid intake of queens before workers emerged.

**
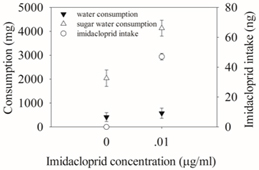
**

**Supplementary Figure 2.** Water and sugar water consumption and imidacloprid intake of incipient colonies during 6 weeks after the first group of workers were emerged.

**
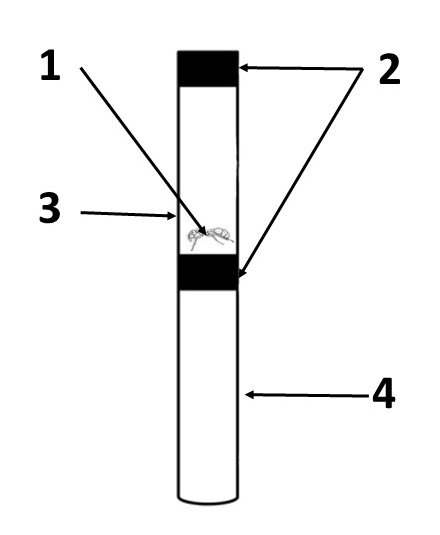
**

**Supplementary Figure 3.** Illustration of the artificial queen chamber.

1. Queen, 2. Cotton balls, 3. Compartment for the queen, 4. Compartment for control or imidacloprid treated water.
